# Characterization of a Novel Reassortant Epizootic Hemorrhagic Disease Virus Serotype 6 Strain Isolated from Diseased White-Tailed Deer (*Odocoileus virginianus*) on a Florida Farm

**DOI:** 10.3390/v14051012

**Published:** 2022-05-10

**Authors:** Thaís C. S. Rodrigues, Pedro H. O. Viadanna, Kuttichantran Subramaniam, Ian K. Hawkins, Albert B. Jeon, Julia C. Loeb, Juan M. C. Krauer, John A. Lednicky, Samantha M. Wisely, Thomas B. Waltzek

**Affiliations:** 1 Department of Infectious Diseases and Immunology, College of Veterinary Medicine, University of Florida, Gainesville, FL 32611, USA; thaiscarneiro_25@hotmail.com (T.C.S.R.); pedro@ufl.edu (P.H.O.V.); kuttichantran@ufl.edu (K.S.); 2 Emerging Pathogens Institute, University of Florida, Gainesville, FL 32611, USA; jloeb@phhp.ufl.edu (J.C.L.); jlednicky@phhp.ufl.edu (J.A.L.); wisely@ufl.edu (S.M.W.); 3 Department of Comparative, Diagnostic, and Population Medicine, College of Veterinary Medicine, University of Florida, Gainesville, FL 32611, USA; iankhawkins@ufl.edu (I.K.H.); abyj85@gmail.com (A.B.J.); 4 Department of Environmental and Global Health, College of Public Health and Health Professions, University of Florida, Gainesville, FL 32611, USA; 5 Department of Large Animal Clinical Sciences, College of Veterinary Medicine, University of Florida, Gainesville, FL 32611, USA; jmcampos@ufl.edu; 6 Department of Wildlife Ecology and Conservation, University of Florida, Gainesville, FL 32611, USA

**Keywords:** Cervidae, epizootic hemorrhagic disease virus, white-tailed deer, disease, phylogeny, orbivirus, reovirus, wildlife

## Abstract

We report an outbreak of a novel reassortant epizootic hemorrhagic disease virus serotype 6 (EHDV-6) in white-tailed deer (WTD) on a Florida farm in 2019. At necropsy, most animals exhibited hemorrhagic lesions in the lung and heart, and congestion in the lung, liver, and spleen. Histopathology revealed multi-organ hemorrhage and congestion, and renal tubular necrosis. Tissues were screened by RT-qPCR and all animals tested positive for EHDV. Tissues were processed for virus isolation and next-generation sequencing was performed on cDNA libraries generated from the RNA extracts of cultures displaying cytopathic effects. Six isolates yielded nearly identical complete genome sequences of a novel U.S. EHDV-6 strain. Genetic and phylogenetic analyses revealed the novel strain to be most closely related to a reassortant EHDV-6 strain isolated from cattle in Trinidad and both strains received segment 4 from an Australian EHDV-2 strain. The novel U.S. EHDV-6 strain is unique in that it acquired segment 8 from an Australian EHDV-8 strain. An RNAscope^®^ in situ hybridization assay was developed against the novel U.S. EHDV-6 strain and labeling was detected within lesions of the heart, kidney, liver, and lung. These data support the novel U.S. reassortant EHDV-6 strain as the cause of disease in the farmed WTD.

## 1. Introduction

Deer farming is a growing industry in the U.S. that generates a direct revenue of approximately 50 million dollars annually [1]. The farmed cervid species include white-tailed deer (WTD, *Odocoileus virginianus*), mule deer (*Odocoileus hemionus*), North American elk or wapiti (*Cervus canadensis*), reindeer (*Rangifer tarandus*), red deer (*Cervus elaphus*), Sika deer (*Cervus nippon*), fallow deer (*Dama dama*), and axis deer (*Axis axis*) [2] (nadefa.org (accessed on 29 March 2022)). Of these, WTD are the most commonly farmed deer species in the U.S. [3].

In Florida, multiple orbiviruses have been isolated from diseased WTD on farms including Big Cypress orbivirus, bluetongue virus (BTV), CHeRI orbiviruses 1–3, epizootic hemorrhagic disease virus (EHDV) serotypes 1, 2, and 6, mobuck orbivirus, and Yunnan orbivirus [4,5,6,7,8,9,10,11]. EHDV is widely distributed among farmed WTD in Florida. A passive surveillance study for EHDV in post-mortem specimens from 55 privately owned deer farms in 30 out of 67 Florida counties between 2016 and 2020 revealed that 27.8% of the sampled WTD were positive by RT-qPCR, and 27.3% of these positives were determined to be EHDV-6 by RT-PCR [12].

EHDV is widely distributed in various temperate and tropical regions of North America, South America, Australia, Asia, and Africa [13,14,15,16]. It is transmitted by biting midges of the genus *Culicoides* (order Diptera, family Ceratopogonidae) to a variety of domestic and wild ungulates of the families Cervidae and Bovidae [17,18,19,20]. EHDV outbreaks have caused significant economic losses, especially in WTD in the U.S. [15,21,22,23]. WTD are known to be highly susceptible to EHDV [23]. Between 2013 and 2014, EHDV was estimated to have killed more than 1.7% of the total farmed cervid population in the U.S. [2]. Clinical signs of this disease in WTD vary from pyrexia, subtle redness of thinly haired regions, progressing to mild depression and lethargy, and in severe cases, severe depression and lethargy, dehydration, subcutaneous swelling around the head, and bleeding [24]. Serotypes 1, 2, 6, and 7 result in similar disease manifestations in WTD [21,24,25,26].

The pathogenesis of EHDV and related orbiviruses involves the transmission of the virus by the bite of infected *Culicoides* spp., followed by replication in mononuclear phagocytes at regional lymph nodes and the release of various cytokines [24,27,28,29]. The virus also replicates in endothelial cells, inducing microvascular damage, platelet aggregation, intravascular thrombosis, disseminated intravascular coagulation, anemia, and thrombocytopenia [24]. The resulting vascular damage can manifest as multisystemic hemorrhage, pulmonary edema, pleural effusion, splenomegaly, ascites, infarction, and tissue necrosis. EHDV and BTV induce similar pathogeneses and are indistinguishable based on clinical signs or post-mortem examination in WTD [23].

Although EHDV infections in cattle are typically self-limiting, those caused by specific serotypes have resulted in significant economic losses. For example, an EHDV-7 outbreak in 2006 resulted in a $2.5 million production loss to the dairy cattle industry in Israel [30,31]. Other economically significant events in cattle include EHDV-6 outbreaks that have occurred in the French island of Réunion (2003 and 2009), Morocco (2004 and 2006), Algeria (2006), Turkey (2007), and Israel (2015) [32,33,34,35,36,37]. Because of the threat posed by EHDV to deer and cattle, it is listed as a reportable disease to the World Organization for Animal Health [37].

EHDV and related orbiviruses possesses 10 linear double-stranded RNA genome segments that encode seven structural (VP1-VP7) and four nonstructural (NS1, NS2, NS3/3a, and NS4) proteins [38,39]. The outer layer of the virion is composed of VP2 and VP5 trimers; these two proteins are the most variable among the EHDV proteins [40]. The interactions of VP2 and VP5 with neutralizing antibodies is used to define the seven EHDV serotypes [13,40]. The internal layers of the virion consist of the immunodominant outer core layer (VP7) and an inner subcore layer (VP3), in which the former is involved in attachment of BTV to the midgut cells of *Culicoides* [41]. Entrapped by the inner subcore layer is the virus core composed of VP1, VP4, and VP6 as well as the ten linear dsRNA segments. The VP1 gene has the largest coding length and encodes the viral RNA-dependent RNA polymerase (RdRp) [42,43,44]. The VP4 gene encodes a guanylyltransferase that acts as a capping enzyme [45,46], and the VP6 gene encodes a helicase [47,48]. The NS1 gene encodes a protein that forms tubules that become attached to the intermediate filaments of the cytoskeleton, and it is highly expressed in the initial hours of infection [49,50]. The NS2 gene encodes an ATPase that facilitates RNA packaging and translation and forms the viral inclusion body [51,52,53,54,55]. The NS3 gene encodes a cell membrane-associated protein involved in virus release from infected cells and is highly expressed in insect cells [56,57,58,59]. The NS4 gene overlaps with the VP6 gene on segment 9 and encodes a nonstructural protein that may confer a replication advantage to BTV by counteracting host antiviral defenses [38,60].

Historically, EHDV serotypes 1 and 2 have been detected throughout wild WTD populations of North America [21,23]. In 2006, an EHDV serotype 6 strain was isolated for the first time from moribund and dead WTD in Indiana and Illinois [22]. From 2006 to 2015, EHDV serotype 6 was isolated from WTD in 16 additional states (including Florida) and is now considered endemic throughout the central and eastern U.S. [4,15,22]. In recent years, EHDV-6 infections have surpassed EHDV-1 in WTD in the country [15]. Comparative genomic analyses of these U.S. EHDV-6 strains (hereafter referred to as U.S. endemic reassortant EHDV-6 strain (Indiana)) revealed that they represent reassortants, with segments 2 and 6 (encoding the serotype determining VP2 and VP5) derived from an exotic EHDV-6 strain (CSIRO 753) and the remaining eight segments derived from a U.S. endemic EHDV-2 strain [22,61]. The exotic EHDV-6 strain (CSIRO 753) was isolated from sentinel cattle in Australia (hereafter referred to as the Australian prototype EHDV-6 strain (CSIRO 753)) [62]. Later studies revealed that another reassortant EHDV-6 strain was circulating in cattle from Trinidad (hereafter referred to as the Trinidad reassortant EHDV-6 strain) [16,63]. The Trinidad reassortant EHDV-6 strain was argued to be the product of assortment events involving the Australian prototype EHDV-6 strain (8 segments), an Australian EHDV serotype 2 strain (segment 4 encoding the VP4 gene), and an EHDV-1 strain of unknown origin (segment 8 encoding the NS2 gene) [63]. The Trinidad reassortant EHDV-6 strain may also be circulating in cattle from neighboring Caribbean islands (Guadeloupe and Martinique) and the South American mainland (French Guiana, Ecuador) [16,63,64,65]. Although the details of how and when the EHDV-6 serotype arrived in the Western Hemisphere remain obscure, it has been suggested that an Australian prototype EHDV-6 strain may have first arrived in the Caribbean and then spread to the U.S. where it donated segments 2 and 6 (encoding VP2 and VP5) in the creation of the U.S. endemic reassortant EHDV-6 strain now circulating in WTD populations in the central and eastern U.S. [15,22,61]. To date, neither the Australian prototype EHDV-6 strain nor the closely related Trinidad reassortant EHDV-6 strain have been detected in the U.S. in either WTD or cattle populations [15,22,61].

In this study, we report the isolation and genomic characterization of the same novel reassortant EHDV-6 strain from dead WTD on a Florida farm in 2019. The observed gross and microscopic pathology and other ancillary diagnostic results supported EHDV-6 as the likely cause of the observed disease in the farmed WTD. A manual RNAscope^®^ in situ hybridization assay targeting the VP1 gene demonstrated labeling of EHDV-6 nucleic acid in the heart, kidney, liver, and lung tissues. Genetic and phylogenetic analyses were conducted to compare this U.S. novel reassortant EHDV-6 strain from WTD to other EHDV strains including EHDV-6 strains isolated from WTD (e.g., U.S. endemic reassortant EHDV-6 strain) and cattle (e.g., Trinidad reassortant EHDV-6 strain and Australian prototype EHDV-6 strain). This study was conducted as part of the University of Florida (UF) Cervidae Health Research Initiative (CHeRI) program, aiming to characterize pathogens negatively impacting farmed WTD in Florida.

## 2. Materials and Methods

### 2.1. Outbreak, Clinical History, and Sample Collection

From 11 September 2019 to 9 November 2019, 12 WTD died or were euthanized on a farm located in Lake County, FL, U.S. The carcasses were necropsied, and each animal was individually identified with a unique number and the prefix “OV”, which denotes *O. virginianus*. The clinical information for each animal is provided in Table 1.

Necropsies were performed by deer farmers or UF technicians following guidelines provided by the CHeRI (https://wec.ifas.ufl.edu/cheri/diagnostics/ (accessed on 29 March 2022)). During the field necropsies, samples of the heart, kidney, liver, lung, spleen, and whole blood were collected and transported on ice prior to fixing in 10% neutral-buffered formalin for histologic processing and/or freezing at −80 °C for diagnostic virology. Lung tissues from animals OV1224, OV1248, OV1289, OV1296, OV1300, OV1314, OV1317, and OV1324 were collected and submitted to the UF Microbiology, Parasitology, and Serology Diagnostic Laboratory of the College of Veterinary Medicine for bacterial and mycological isolation and identification. Samples of the heart, kidney, liver, lung, small intestine (only available for animal OV1248), and spleen from animals OV1248 and OV1296 were fixed in 10% neutral-buffered formalin and submitted for histologic processing and histopathologic examination at the UF Veterinary Diagnostic Laboratories. Following routine processing, 3-µm sections of the formalin-fixed, paraffin-embedded tissue samples (listed above) were stained with hematoxylin and eosin (H&E). This work was approved by the UF Institutional Animal Care and Use Committee (IACUC Protocol Numbers 201609390 and 201909390).

### 2.2. RT-PCR Detection of BTV, EEEV, EHDV, and WNV vRNA

Prior to extracting virus RNA (vRNA) from the heart, kidney, liver, lungs, and spleen samples, the tissues (previously stored at −80 °C) were thawed, aseptically minced using forceps, then homogenized to generate 10% *w*/*v* cell-free suspension homogenates in sterile phosphate-buffered saline (PBS) using a sterile manual tissue grinder (Fisher Scientific, Waltham, MA, USA). The resulting homogenates were subsequently cleared of debris by low-speed centrifugation (5 min at 1500× *g*) and aseptically transferred to sterile polypropylene centrifuge tubes. vRNA was extracted from virions in the tissue homogenates and whole blood samples using a QIAamp Viral RNA Mini kit (Qiagen, Valencia, CA, USA) following the manufacturer’s protocol. RNA extracts from blood and tissue homogenates of all animals were then screened for BTV and EHDV using a multiplex quantitative real-time reverse-transcription polymerase chain reaction (RT-qPCR) assay as previously described [66]. For EHDV-positive homogenates, EHDV serotype was determined using a multiplex RT-PCR assay as previously described [67]. vRNA extracted from spleen homogenates were also tested for eastern equine encephalitis virus (EEEV) and West Nile virus (WNV) using a VetMAX Plus One-Step RT-qPCR kit (Applied Biosystem), as previously described [68,69]. Briefly, 25-µL reactions containing 12.5 µL 2X RT-PCR Buffer, 1 µL 25X RT-PCR enzyme, RNAase-free water, 1 µL Xeno VIC assay primers and probe, and 0.1 µL Xeno RNA (internal positive control), to which was added 25 pmol (for WNV) or 19 pmol (for EEEV) of each primer and 5 pmol of probe: fluorophore 6-FAM, quencher BHQ1, resulting in a 21 µL master-mix. Thereafter, 4 µL of vRNA was added to the master mix containing either WNV or EEEV specific primers and probes, and RT-PCRs were performed in an Applied Biosystems 7500 fast Real-Time PCR System as follows: reverse transcription step at 48 °C for 10 min, initial denaturation step at 95 °C for 10 min followed by 40 cycles of 2-step cycling consisting of 95 °C for 15 s, and annealing/extension at 60 °C for 45 s.

### 2.3. RNAScope^®^ In Situ Hybridization (ISH) Assay

A manual in situ hybridization (ISH) assay using RNAscope^®^ technology was used to detect EHDV-6 RNA in formalin-fixed paraffin-embedded (FFPE) heart, kidney, liver, and lung tissues from animals OV1248 and OV1296. Specific probes were designed by Advanced Cell Diagnostics (Newark, CA, USA), based on the following genes: dihydrodipicolinate reductase gene (dapB; negative control probe) [catalog # 312038]; WTD cytochrome B gene (OV cytB; internal positive control probe) [catalog # 868021]; and the EHDV-6 VP1 gene (EHDV-6 VP1; test probe) [catalog # 868011].

The ISH assay was performed using an RNAscope^®^ 2.5 HD Red Detection Kit (ACD, Hayward, CA) following the protocol provided by the manufacturer [70]. Three sections of 4 μm were cut from each paraffin block and mounted on Fisherbrand SuperFrost Plus glass slides (Fisher Scientific, Pittsburgh, PA, USA). The slides were subjected to deparaffinization, target retrieval, protease digestion, and endogenous enzyme block. Probe hybridization was performed, and each of the three sections of a block was treated with a specific probe: one section received the dapB probe, a second section received the OV cytB probe, and a third section received the EHDV-6 VP1 probe. After probe hybridization, signal amplification, fast red chromogenic development, and counterstaining with hematoxylin was performed. Incubations were conducted in a humidity chamber and a HybEZTM oven (ACD, Hayward, CA, USA). After the slides were dried and mounted, they were examined for microscopic lesions using an Olympus BX53 light microscope (Olympus, Center Valley, PA, USA).

### 2.4. Virus Isolation in Cultured Cells

Virus isolation was attempted using the *Aedes albopictus* (Asian tiger mosquito) cell line C6/36 (ATCC CRL1660) for all animals except OV1299 as previously described [4]. Spleen samples were thawed and homogenized in sterile phosphate-buffered saline (PBS), using a sterile manual tissue grinder (Fisher Scientific) to generate 10% *w*/*v* cell-free homogenates. The suspension was cleared of debris by low-speed centrifugation (5 min at 1500× *g*). The supernatant was then filtered through a sterile 0.45 µm pore-size polyvinylidene fluoride filter (Fisher Scientific, Cat. Number 09-720-4). A 0.5 mL aliquot of the filtrate was inoculated onto confluent C6/36 cells in 25 cm^2^ culture flasks. The cells were re-fed every 3 days and observed daily for virus-induced cytopathic effects (CPE) for 30 days. Non-inoculated cells were maintained in parallel as negative controls. When no CPE was observed by day 30 post-inoculation, a second passage was performed, and the cells were observed for another 30 days before being considered negative for virus isolation. After CPE were observed in 50% of the infected cells, they were scraped and harvested along with the spent cell growth medium and stored at −80 °C for further analyses.

### 2.5. Next-Generation Sequencing (NGS)

The frozen spent cell-growth media from samples were thawed and spun to remove cellular debris prior to extracting vRNA from virions using a QIAamp Viral RNA Mini kit (Qiagen) according to the manufacturer’s protocol. cDNA libraries were generated using a NEBNext Ultra RNA Library Prep kit (Illumina, San Diego, CA, USA) and sequenced on an Illumina MiSeq sequencer (Illumina). Host (*Aedes albopictus*) sequences (GenBank accession number MNAF00000000.2) were removed using Kraken v2.0 (Johns Hopkins University School of Medicine, Baltimore, MD, USA) [71]. After removing the host sequences, the de novo assembly of the remaining paired-end reads was performed using SPAdes 3.5.0 [72]. The assembled contigs were then subjected to BLASTX searches against the National Center for Biotechnology Information (NCBI) nonredundant protein database, using OmicsBox v1.2.

### 2.6. Phylogenetic and Genetic Analysis

Maximum Likelihood (ML) phylogenetic trees were constructed based on separate nucleotide alignments of each of the 10 coding sequences of the EHDV isolated from OV1321 to 66 other EHDVs and one BTV (outgroup), whose sequences were available in the NCBI GenBank database (Supplemental Table S1). The alignments were performed using the MAFFT server (https://mafft.cbrc.jp/alignment/server/ (accessed on 29 March 2022)) [73], and the ML trees were constructed in IQ-TREE with 1000 non-parametric standard bootstraps performed to test the robustness of the clades [74].

## 3. Results

### 3.1. Necropsy Findings, Bacterial Isolation, Histopathology, and RNAscope^®^ In Situ Hybridization Assay

**Figure 1:**
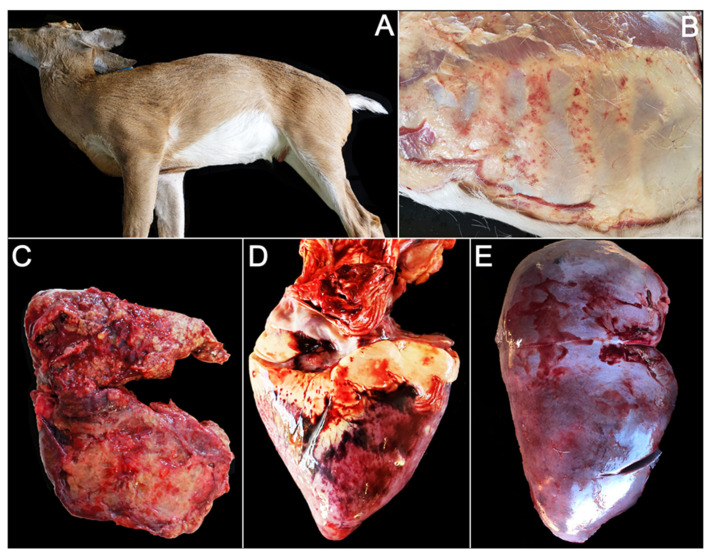
Gross photos of farmed white-tailed deer naturally infected with the U.S. novel reassortant EHDV-6 strain. (**A**) OV1296: swelling of the neck and jaw; (**B**) OV1300: diffuse, moderate petechiae of the thoracic and abdominal fascia; (**C**) OV1324: pulmonary hemorrhage with concurrent suppurative pneumonia; (**D**) OV1300: multifocal epicardial hemorrhage; (**E**) OV1265: hepatic congestion.

Two animals exhibited swelling of the neck, and two other animals exhibit swelling of the tongue (Table 1, Figure 1A). Animal OV1300 showed diffuse, moderate petechia of the thoracic and abdominal fascia (Figure 1B). Ten animals exhibited signs of pulmonary congestion and/or hemorrhage. One animal (OV1324) presented with severe suppurative pneumonia (Figure 1C); cultures yielding both Gram-negative (*Proteus* sp., *Pseudomonas* sp.) and Gram-positive (*Streptococcus* sp.) bacteria. *Escherichia coli* and other members of the family Enterobacteriaceae were isolated from the lungs of 3/12 animals (Table 2). The most common cardiac lesion was hemorrhage (9/12), ranging from mild petechia (6/12) to large, multifocal ecchymoses (1/12) (Figure 1D). Congestion of the liver (8/12) and spleen (7/12) was commonly observed (Figure 1E, Table 2).

**Figure 2:**
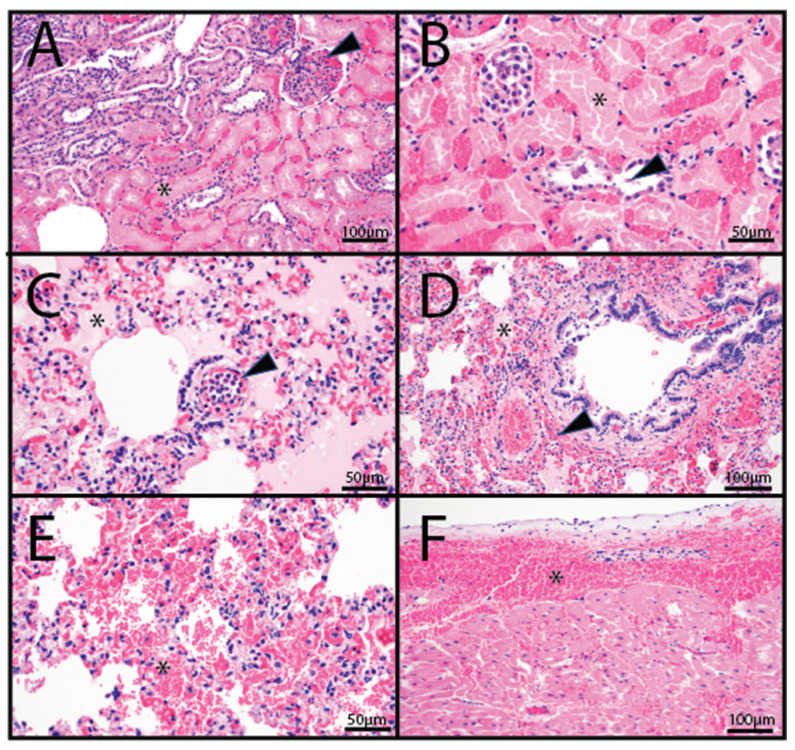
Histopathology photomicrographs of farmed white-tailed deer naturally infected with the U.S. novel reassortant EHDV-6 strain. (**A**) OV1248; kidney: large areas of coagulative tubular necrosis (asterisk) with interstitial and glomerular congestion (arrowhead). (**B**) OV1248; kidney: occasional tubules within necrotic areas contained accumulations of a basophilic to eosinophilic material admixed with individualized exfoliated epithelial cells (arrowhead). (**C**) OV1296; lung: vascular congestion and pulmonary edema (asterisk); numerous intravascular to marginated neutrophils (arrowhead). (**D**) OV1296; lung: pulmonary edema (asterisk) and perivascular and peribronchiolar hemorrhage (arrowhead). (**E**) OV1296; lung: pulmonary hemorrhage (asterisk). (**F**) OV1296; heart: subepicardial hemorrhage (asterisk).

Histopathology was performed on tissues from two animals (OV1248 and OV1296). Both animals exhibited mild to moderate hepatic congestion, and variable pulmonary congestion with edema. Animal OV1248 exhibited multifocal, acute, moderate, coagulative necrosis of tubular epithelial cells with associated interstitial hemorrhage and congestion in the kidneys (Figure 2A,B). The interalveolar septa were often infiltrated by low numbers of neutrophils, and small to medium caliber pulmonary vessels occasionally contained medium numbers of intraluminal and marginated neutrophils (Figure 2C). Additionally, the lungs of animal OV1296 exhibited acute, multifocal, moderate hemorrhage (Figure 2D,E) and moderate, multifocal, submucosal hemorrhage of the bronchi. In the heart, there was moderate, multifocal, subepicardial hemorrhage, elevating the epicardium and extending into the myocardium (Figure 2F). The spleen exhibited moderate, multifocal hemorrhage.

**Figure 3:**
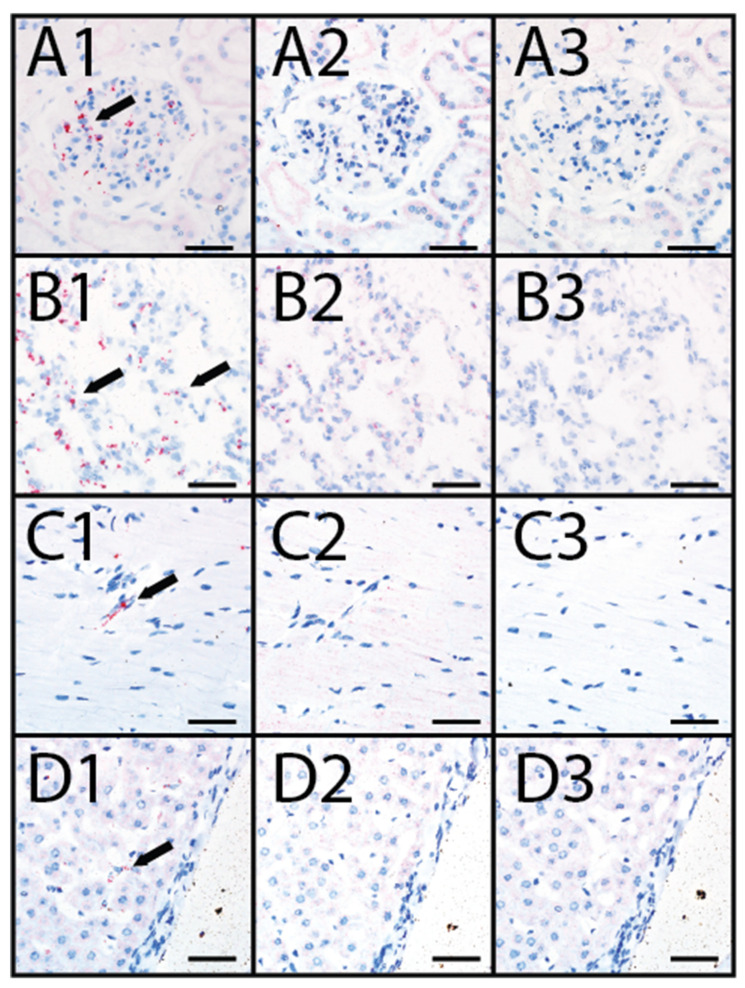
RNAscope^®^ in situ hybridization (ISH) in farmed deer naturally infected with the U.S. novel reassortant EHDV-6 strain. (**A1**) OV1248, kidney processed with the EHDV probe: positive EHDV nucleic acid red labeling (black arrow) within endothelium of the glomerulus; (**A2**) OV1248, kidney processed with the WTD cytB internal positive control probe: positive red labeling is evident in renal interstitial cells; (**A3**) OV1248, kidney processed with the dapB negative control probe: no red labeling detected; (**B1**) OV1248, lung processed with the EHDV probe: positive EHDV nucleic acid red labeling (black arrow) detected within pneumocytes and macrophages; (**B2**) OV1248, lung processed with the WTD cytB positive control probe: positive red labeling detected in pulmonary interstitial cells; (**B3**) OV1248, lung processed with the dapB negative control probe: no red labeling detected; (**C1**) OV1248, heart processed with the EHDV probe: positive EHDV nucleic acid red labeling (black arrow) detected within vascular endothelium; (**C2**) OV1248, heart processed with the WTD cytB positive control probe: red labeling detected in cardiac myocytes; (**C3**) OV1248, heart processed with the dapB negative control probe: no red labeling detected; (**D1**) OV1248, liver processed with the EHDV probe: positive EHDV nucleic acid red labeling (black arrow) detected within sinusoidal endothelium; (**D2**) OV1248, liver processed with the WTD cytB positive control probe: positive red labeling detected in hepatocytes; (**D3**) OV1248, liver processed with the dapB negative control probe: no red labeling detected. Scale bars = 50 µm.

Heart, kidney, liver, and lung tissue sections from animals OV1248 and OV1296 were evaluated for EHDV-6 RNA using RNAScope^®^ in situ hybridization. EHDV-6 RNA was detected within scattered cells of the heart, kidney, liver, and lung (Figure 3). Distribution of EHDV-6 labeling was most prominent in endothelial cells, especially in renal glomerular capillaries (Figure 3(A1)), alveolar capillaries of the lung (Figure 3(B1)), small myocardial vessels (Figure 3(C1)), and endothelial lining of the hepatic sinusoids (Figure 3(D1)). EHDV-6 labeling was occasionally observed within alveolar macrophages. The CytB probe (internal positive control) displayed labeling in the evaluated tissues (Figure 3(A2,B2,C2,D2)). No staining was observed when the dapB (negative control) probe was applied to the tissue sections (Figure 3(A3,B3,C3,D3)).

### 3.2. RT-PCR Detection of BTV, EEEV, EHDV, and WNV vRNA

RNA extracts from one or more tissues from all 12 animals were positive for EHDV by RT-qPCR (Table 3). Spleen RNA extracts from six animals were positive for EHDV-6, one deer was positive for EHDV-2, and one was positive for both EHDV-2 and EHDV-6. Four animals yielded inconclusive results using the EHDV typing RT-PCR assay and were classified as “untypeable”. The serotype of sample OV1288 was determined using the next-generation sequencing data (described below in Section 3.4), and it was classified as EHDV-6 based on its VP2 and VP5 gene sequences. All spleen vRNA extracts were negative for EEEV and WNV. The only animal positive for BTV was OV1299.

### 3.3. Virus Isolation

After 5 days post-inoculation, formation of cytoplasmic inclusions concomitant with granulation of the cytoplasm, evolving to enlargement of cells, and subsequent detachment from the growing surface were observed for samples OV1224, OV1248, OV1265, OV1288, OV1289, OV1296, OV1300, OV1314, and OV1321. No CPE was observed for samples OV1317 and OV1324 after two passages and they were considered negative for virus isolation.

### 3.4. Genome Sequencing, Phylogenetic, and Genetic Analysis

Seven samples that displayed CPE in C6/C36 cell cultures (OV1224, OV1248, OV1265, OV1288, OV1296, OV1314, and OV1321) were analyzed by NGS. Samples OV1289 and OV1300 were not included in the NGS analysis due to low virus copy numbers present in the clarified spent cell-growth media (data not shown). De novo assembly of sample OV1321 resulted in the complete gene coding sequences for all 10 segments of the genome (GenBank accession nos. OK106265-OK106274). The genomic sequences for samples OV1296 and OV1314 were not submitted to GenBank because they were 100% identical to sample OV1321. Samples OV1248 (accession nos. OK500217-OK500226), OV1265 (accession nos. OK500227-OK500236), and OV1288 (accession nos. OK500237-OK500246) resulted in the complete gene coding sequences for all segments, except for the first one to four initial nucleotides of segment 4. Samples OV1296 and OV1314 resulted in the complete gene coding sequence for all segments, except for the four initial nucleotides of segments 1 and 4. For sample OV1224, the coding sequence for six or more segments were incomplete, and thus, it was excluded from the analysis. The coding sequences of OV1248, OV1265, and OV1288 were nearly identical to OV1321, OV1296, and OV1314 (99.85% to 99.97%) (Table 4). The OV1321 coding sequences for segments 1, 2, 3, 6, 9, and 10 demonstrated the next highest nucleotide identities (96.89–98.03%) to an Australian prototype EHDV-6 strain (CSIRO 753) (Table 4). For segments 2 and 6, OV1321 displayed 96.57 and 96.4% identity to the U.S. endemic reassortant EHDV-6 strain (Indiana), respectively (Table 4). The OV1321 coding sequences for segments 4, 5, and 7 demonstrated the next highest nucleotide identities (97.53–97.73%) to the Trinidad reassortant EHDV-6 strain. For segment 4, OV1321 displayed 96.23% identity to an Australian EHDV-2 strain (CSIRO 439). The OV1321 coding sequence for segment 8 displayed the highest nucleotide identity (91.76%) to an EHDV-8 strain (CPR 3961A), isolated from overtly healthy sentinel cattle in Australia in 1982 (Table 4). For segment 10, OV1321 displayed 97.53% identity to both the Trinidad reassortant EHDV-6 strain and the Australian prototype EHDV-6 strain (CSIRO 753). Hereafter, the EHDV-6 isolate from animal OV1321 is referred to as the U.S. novel reassortant EHDV-6 strain.

**Figure 4:**
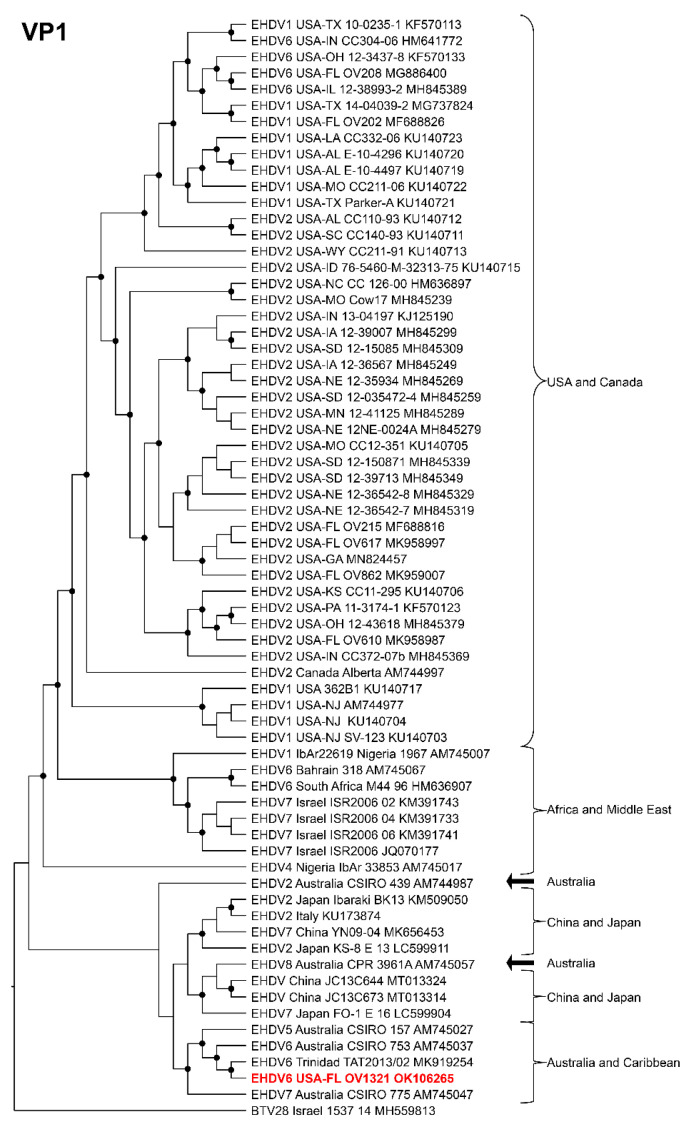
Maximum Likelihood cladogram depicting the relationships of the U.S. novel reassortant EHDV-6 strain isolated from a white-tailed deer (OV1321) to 66 other EHDV strains. The tree was generated from the nucleotide sequence alignment of the VP1 gene. The included EHDV and BTV strains are indicated by serotype, country, state (for U.S. strains), strain or isolate name, and GenBank accession no. Nodes with black circles are supported by bootstrap values ≥80%. The tree was rooted with the BTV-28 strain. Additional metadata for each virus in the tree are provided in Supplemental Table S1.

**Figure 5:**
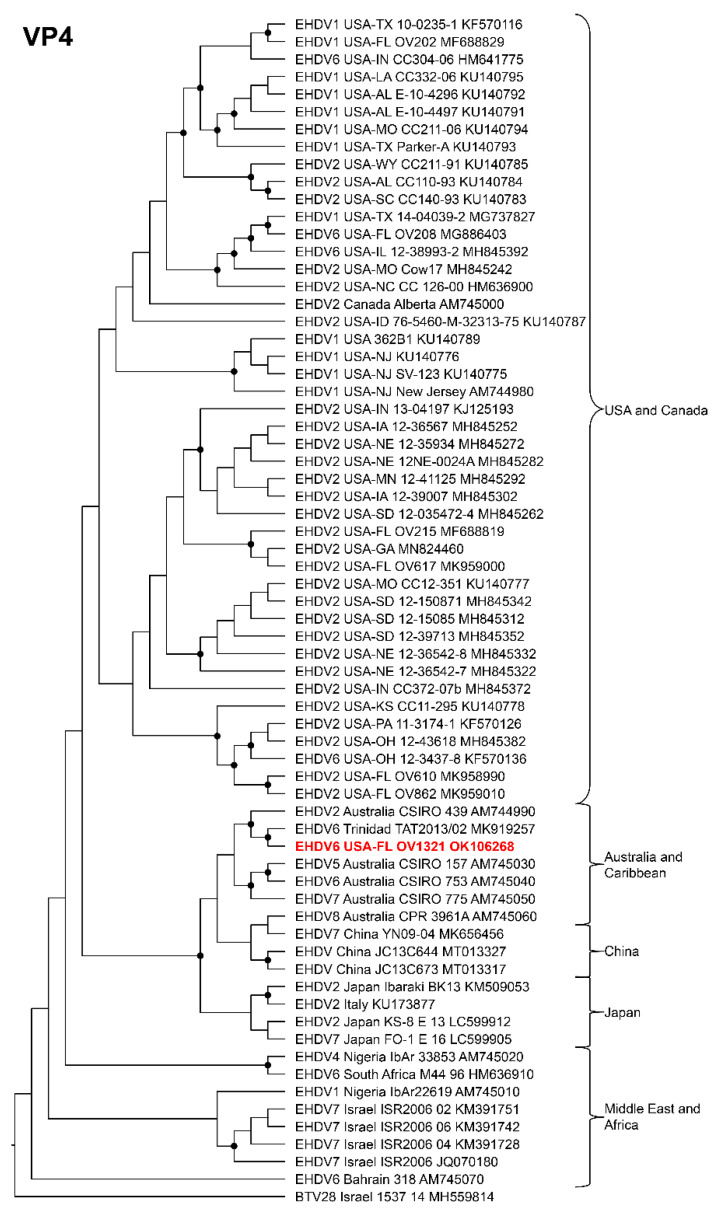
Maximum Likelihood cladogram depicting the relationships of the U.S. novel reassortant EHDV-6 strain isolated from a white-tailed deer (OV1321) to 66 other EHDV strains. The tree was generated from the nucleotide sequence alignment of the VP4 gene. The included EHDV and BTV strains are indicated by serotype, country, state (for U.S. strains), strain or isolate name, and GenBank accession no. Nodes with black circles are supported by bootstrap values ≥80%. The tree was rooted with the BTV-28 strain. Additional metadata for each virus in the tree are provided in Supplemental Table S1.

**Figure 6:**
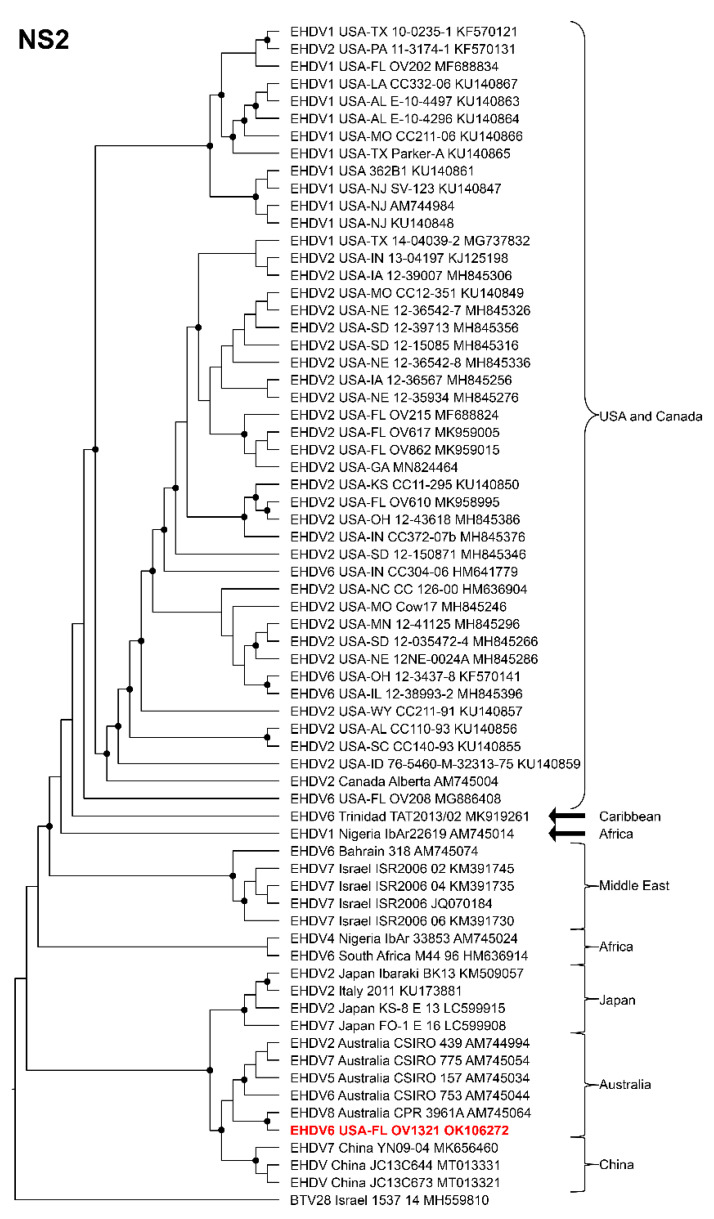
Maximum Likelihood cladogram depicting the relationships of the U.S. novel reassortant EHDV-6 strain isolated from a white-tailed deer (OV1321) to 66 other EHDV strains. The tree was generated from the nucleotide sequence alignment of the NS2 gene. The included EHDV and BTV strains are indicated by serotype, country, state (for U.S. strains), strain or isolate name, and GenBank accession no. Nodes with black circles are supported by bootstrap values ≥80%. The tree was rooted with the BTV-28 strain. Additional metadata for each virus in the tree are provided in Supplemental Table S1.

**Figure 7:**
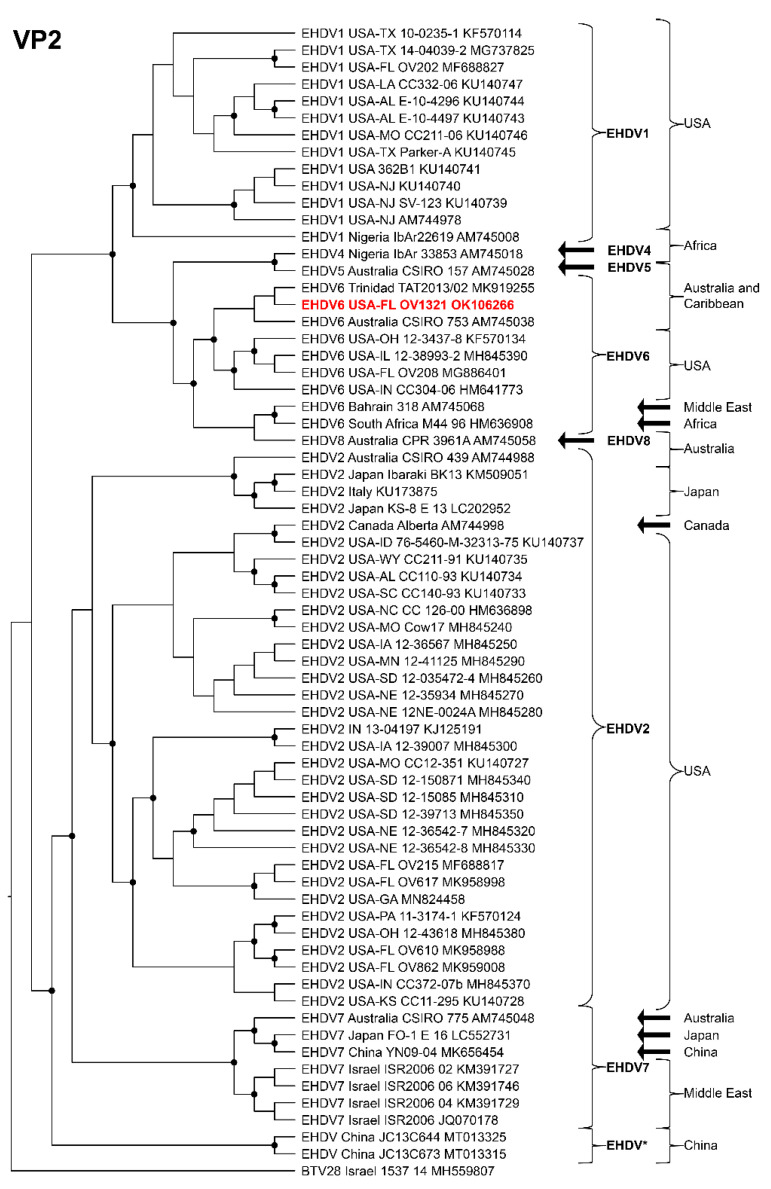
Maximum Likelihood cladogram depicting the relationships of the U.S. novel reassortant EHDV-6 strain isolated from a white-tailed deer (OV1321) to 66 other EHDV strains. The tree was generated from the nucleotide sequence alignment of the VP2 gene. The included EHDV and BTV strains are indicated by serotype, country, state (for U.S. strains), strain or isolate name, and GenBank accession no. Nodes with black circles are supported by bootstrap values ≥80%. The tree was rooted with the BTV-28 strain. Additional metadata for each virus in the tree are provided in Supplemental Table S1.

Phylogenetic analyses based on the coding sequences of the VP1, VP3, VP4, VP6, NS1, and NS3 genes supported the U.S. novel reassortant EHDV-6 strain (OV1321) as the sister group to the Trinidad reassortant EHDV-6 strain (Figure 4 and Figure 5, Supplemental Figures S1–S4). The same gene trees, except VP4, supported the Australian prototype EHDV-6 strain (CSIRO 753) as the sister group to the clade formed by the aforementioned reassortant EHDV-6 strains from the U.S. and Trinidad. The VP4 gene analysis supported an EHDV-2 strain from Australia (CSIRO 439) as the sister group to the clade formed by the aforementioned reassortant EHDV-6 strains from the U.S. (novel) and Trinidad. The NS2 gene tree supported the U.S. novel reassortant EHDV-6 strain (OV1321) as the sister group to an EHDV-8 strain (CPR 3961A) from Australia (Figure 6). The VP2 and VP5 gene analyses supported a clade composed of the Australian prototype EHDV-6 strain (CSIRO 753), Trinidad reassortant EHDV-6 strain, the U.S. novel reassortant EHDV-6 strain (OV1321), and the U.S. endemic reassortant EHDV-6 strains (Indiana 12-3437-8, Illinois 12-38993-2, Indiana CC304-06, Ohio 12-3437-8, Florida OV208) (Figure 7 and Supplemental Figure S5). These two gene trees revealed the close relationship of the U.S. endemic reassortant EHDV-6 strains to each other; however, their relationship to the other EHDV-6 strains was not resolved. The VP7 gene analysis supported the Australian prototype EHDV-6 strain (CSIRO 753), the Trinidad reassortant EHDV-6 strain, and the U.S. novel reassortant EHDV-6 strain (OV1321) as a clade but could not resolve the relationships of these viruses to each other (Supplemental Figure S6). In summary, genetic and phylogenetic analyses revealed the close phylogenetic relationship of the U.S. novel reassortant EHDV-6 strain (OV1321) with the Trinidad reassortant EHDV-6 strain and the Australian prototype EHDV-6 strain (CSIRO 753).

## 4. Discussion

EHDV serotypes 1, 2, and 6 are endemic in farmed and free-ranging WTD populations in North America [21,23]. Our study confirmed an outbreak of an EHDV-6 strain in farmed WTD in Florida in 2019 based on clinical signs, gross and microscopic pathology, in situ hybridization (ISH), RT-PCR and RT-qPCR, virus isolation, and next-generation sequencing. Phylogenetic and genetic analyses of the coding sequences of six Florida EHDV isolates supported them as a novel U.S. reassortant EHDV-6 strain (OV1321).

The gross and microscopic lesions associated with the Florida EHDV-6 outbreak in farmed WTD in 2019 were similar to those previously described for an experimental infection of EHDV-6 in WTD [26] including: (1) widespread hemorrhages on the abdominal muscle fascia, epicardium, and rumen wall; (2) mild to moderate hepatic congestion; (3) varying levels of pulmonary congestion, hemorrhage, and edema; (4) moderate subepicardial hemorrhage; and (5) moderate coagulative necrosis with associated interstitial hemorrhage and congestion of the kidneys. The EHDV-6 ISH labeling appeared subjectively greatest in cells morphologically resembling macrophages in the lung and liver parenchyma and the endothelium of all organs evaluated. These results are consistent with the monocytotropic and endotheliotropic nature of EHDV [26,75,76]. The prominent labeling of endothelium likely explains the vascular compromise and resulting multisystemic hemorrhage, pulmonary edema, and renal tubular coagulative necrosis. Similar to the intense staining of the lung sections by ISH, the RT-qPCR data supported high EHDV loads in the lungs (Ct values of 22 and 25 in OV1248 and OV1296, respectively). Taken together, the observed pathological findings and the results of the virus culture and molecular assays supported EHDV-6 as the cause of disease in the farmed WTD.

Genetic and phylogenetic analyses of the majority of the structural and non-structural genes supported the close relationship of the U.S. reassortant EHDV-6 strain (OV1321) to the Trinidad reassortant EHDV-6 strain and confirms the progenitor of these Western Hemisphere EHDV-6 strains to be an Australian EHDV-6 strain (CSIRO 753). The closely related U.S. reassortant EHDV-6 strain (OV1321) and the Trinidad reassortant EHDV-6 strain are unique among EHDV-6 strains in that they both received segment 4 from an Australian EHDV-2 strain (CSIRO 439) [63]. The U.S. reassortant EHDV-6 strain (OV1321) and the Trinidad reassortant EHDV-6 strain can be differentiated from each other in that the former possesses an NS2 gene derived from an Australian EHDV-8 strain (CPR 3961A) and the latter received its NS2 gene after it arrived in the Western Hemisphere from a yet unknown EHDV strain [63].

Previous phylogenetic analyses of the U.S. endemic reassortant EHDV-6 strain (Indiana) from diseased WTD in the central and eastern U.S. since 2006 revealed it evolved through reassortment with EHDV-6 strains from Australian cattle and a EHDV-2 strain from U.S. WTD [16,22,63]. The serotype determining outer capsid proteins (VP2 and VP5) of the U.S. endemic reassortant strain (Indiana) were derived from an Australian prototype EHDV-6 strain (CSIRO 753) and the remaining gene segments encoding both nonstructural and structural proteins derived from a U.S. EHDV-2 strain [16,22,63]. However, the donor of the two EHDV-6 segments in the U.S. endemic reassortant strain (Indiana) remained obscure [22]. Later studies suggested a reassortant EHDV-6 strain isolated from asymptomatic cattle in Trinidad, with 9/10 segments derived from Australian EHDV-6 (CSIRO 753) and EHDV-2 (CSIRO 439) strains, may have spread to the U.S. prior to 2006, where it then served as the donor of the EHDV-6 segments in the creation of the U.S. endemic reassortant EHDV-6 strain (Indiana) [16,63]. Previous phylodynamic analyses estimated that a prototype Australian EHDV-6 strain entered the Western Hemisphere around 1966 [16]. Additional evidence that the EHDV-6 serotype was circulating in the U.S. prior to 2006 is suggested by the detection of neutralizing antibodies against EHDV-6 in WTD in the southeastern U.S. as early as 2000 [77].

It is also possible that the U.S. novel reassortant EHDV-6 strain (OV1321) characterized in our study was present in the U.S. prior to 2006 and it served as the donor of the EHDV-6 segments to the U.S. endemic reassortant EHDV-6 strain (Indiana). However, our phylogenetic analyses, based on the VP2 and VP5 genes, did not resolve the relationship of the U.S. novel reassortant EHDV-6 strain (OV1321) to the other EHDV-6 strains (e.g., U.S. endemic, Australian, and Caribbean EHDV-6 strains). If the U.S. novel reassortant EHDV-6 strain (OV1321) has been present in the U.S. for some time, it is also possible that it spread from the U.S. to the Caribbean via movement of an infected ungulate host (such as cattle) or competent vector [16,63]. Additional complete genome sequences of EHDV-6 strains across a greater range of hosts (e.g., bovids/cervids), years (e.g., prior to 2006), and geographic regions (e.g., U.S., Caribbean, and South America) would facilitate phylodynamic modeling that could result in a better understanding of the timing and number of incursions of EHDV-6 strains into the Western Hemisphere, and their subsequent dissemination within the region.

The EHDV-6 seroprevalence is high in domestic cattle ≤ 2 years of age in Florida [78]. To date, EHDV-6 strains derived from the U.S., Caribbean, and Australia have not resulted in clinical disease in cattle [16,26,62,64]. However, the disease potential of the U.S. novel reassortant EHDV-6 strain (OV1321) in cattle remains to be determined. Future research is needed to determine the susceptibility and potential role in disease of this novel EHDV-6 strain in both domestic and wild ungulates.

## Figures and Tables

**Table 1:** Age, sex, clinical signs, date of death, and date of necropsy of 12 farmed white-tailed deer, Florida, U.S in 2019.

Animal ID	Age (Years)	Sex	Clinical Signs	Date of Death and Necropsy
OV1224	1	female	Separation from the herd, lethargy, ataxia, forelimb abrasions, swollen neck, scleral vascular congestion, palpebral edema, and pale oral mucosa	11 September
OV1248	1	male	Lethargy, ptyalism, hindlimb abrasions, swollen face, and a swollen and ulcerated tongue	14 September
OV1265	2	male	Separation from the herd	23 September *
OV1288	2	female	Recumbent and ptyalism	26 September *
OV1289	1	female	Separation from the herd, lethargy, and recumbent	30 September
OV1296	1	male	Lethargy, mucoid nasal discharge, swelling of the jaw and neck	9 October
OV1299	1	female	No clinical signs noted	4 October *
OV1300	1	female	Animal was recovering from pneumonia when it became lethargic, recumbent, and observed with a swollen togue and pale oral mucosa, thin body condition	11 October **
OV1314	1	male	Ataxia	18 October
OV1317	1	male	Tongue ulceration, severe dehydration, and dyspnea	29 October
OV1321	0.5	male	No clinical signs noted	6 November
OV1324	3	female	Lethargy and thin body condition	9 November

* These animals were found dead. ** This animal was necropsied on 12 October.

**Table 2:** Bacteriology and necropsy findings of the 12 farmed white-tailed deer, Florida, U.S.

Animal ID	Bacterial ID	Abnormalities Observed at Necropsy			
	Lungs	Lungs	Heart	Liver	Spleen
OV1224	*E. coli*/*Enterococcus* sp.	Congestion, edema, hemorrhage	Endocardial petechia	Congested	Congested
OV1248	*E. coli*/*Enterococcus* sp.	Congestion, edema, hemorrhage	Endo/epicardial hemorrhage	Pale	nr
OV1265	Not tested	Congestion, edema, hemorrhage	Epicardial petechia	Congested	Congested
OV1288	Not tested	Congestion, edema, hemorrhage	Epicardial petechia	Congested	Congested
OV1289	*Actinobacter baumannii*	Congested	Pericardial hemorrhage	Congested	Congested
OV1296	*Enterobacter gergoviae*	Congested, hemorrhage	Epicardial petechia	Congested, hemorrhage	Congested
OV1299	Not tested	None	None	None	None
OV1300	*E. coli*/*Proteus* sp.	Congestion, edema, hemorrhage	Epicardial petechia/ecchymosis	Congested	Congested
OV1314	No growth	None	None	None	None
OV1317	*Pseudomonas aeruginosa*/*Enterococcus* sp./*Trueperella pyogenes*	Congested, foaming present in bronchiole, hemorrhage	Epicardial petechia	Congested	None
OV1321	Not tested	Hemorrhage	Endo/epicardial petechia	None	None
OV1324	*Proteus* sp./*Streptococcus* sp./*Pseudomonas* sp.	Congested, hemorrhage, diffuse abscess	nr	Congested	Congested

nr: not recorded.

**Table 3:** Results of the RT-PCR and RT-qPCR testing for EHDV and BTV in tissues and whole blood taken from 12 farmed white-tailed deer, Florida, U.S.

Animal ID	EHDV Typing RT-PCR	EHDV qPCR (Ct Value)						BTV qPCR (Ct Value)	
	Spleen	Heart	Kidney	Liver	Lung	Spleen	Whole Blood	Whole Blood	Spleen
OV1224	EHDV-6	32	34	30	26	30	30	neg	nt
OV1248	EHDV-6	28	27	30	22	28	28	neg	nt
OV1265	EHDV-6	nt	nt	nt	nt	28	nt	nt	neg
OV1288	untypeable	nt	nt	nt	nt	32	nt	nt	neg
OV1289	EHDV-2 & -6	31	34	33	25	30	nt	nt	neg
OV1296	EHDV-6	25	30	26	25	25	nt	nt	neg
OV1299	untypeable	neg	33	39	34	31	nt	nt	32
OV1300	untypeable	37	neg	38	35	35	nt	nt	neg
OV1314	EHDV-6	32	25	29	22	28	nt	nt	neg
OV1317	untypeable	31	nt	neg	35	36	nt	nt	neg
OV1321	EHDV-6	nt	nt	nt	nt	23	nt	nt	neg
OV1324	EHDV-2	nt	nt	nt	nt	38	nt	nt	neg

neg: negative, nt: not tested.

**Table 4:** Nucleotide identities for the coding sequences of each segment of the U.S. novel reassortant EHDV-6 strain isolated from a white-tailed deer (OV1321) compared to other EHDV-6 isolates from the same outbreak (OV1248, OV1265, OV1288, OV1296, and OV1314) and to selected EHDV-2, EHDV-6, and EHDV-8 isolates. Segments with incomplete coding sequences are indicated with the superscript P for partial. The highest nucleotide identities of EHDV-6 (OV1321) compared to selected EHDV-6 and EHDV-8 are bolded. Additional metadata for each EHDV isolate are provided in Supplemental Table S1.

Gene Name	OV1248	OV1265	OV1288	OV1296	OV1314	EHDV-6				EHDV-8	EHDV-2
						TAT2013/02	CSIRO 753	318	Indiana	CPR 3961A	CSIRO 439
*VP1*	100	99.92	100	100^P^	100^P^	96.85	**97.52**	78.43	77.45	93.35	88.21
*VP2*	99.97	100	99.97	100	100	97.05	**97.12**	72.55	96.57	69.06	72.97
*VP3*	100	99.89	99.96	100	100	96.56	**96.89**	79.22	79.37	92.11	94.19
*VP4*	99.95^P^	99.95^P^	100^P^	100^P^	100^P^	**97.73**	91.99	75.30	75.25	91.78	96.23
*NS1*	100	100	100	100	100	**97.70**	97.22	80.62	79.83	86.15	93.95
*VP5*	100	100	100	100	100	96.21	**96.91**	80.24	96.40	86.30	69.02
*VP7*	100	100	100	100	100	**97.71**	97.14	78.76	79.52	79.79	80.00
*NS2*	100	99.91	100	100	100	74.39	79.93	70.93	71.29	**91.76**	79.13
*VP6*	100	100	100	100	100	97.14	**98.03**	76.04	77.81	93.20	93.79
*NS3*	100	99.85	100	100	100	**97.53**	**97.53**	75.98	77.58	77.87	94.26

## Data Availability

The complete gene coding sequences for all 10 segments of the genomes recovered in this study been deposited in the NCBI GenBank database and are available under the GenBank accession nos. OK106265-OK106274 (for OV1321), OK500217-OK500226 (for OV1248), OK500227-OK500236 (for OV1265), and OK500237-OK500246 (for OV1288).

## Supplementary Materials

The following supporting information can be downloaded at: https://www.mdpi.com/article/10.3390/v14051012/s1, Figure S1: Maximum Likelihood cladogram depicting the relationships of the U.S. novel reassortant EHDV-6 stain isolated from a white-tailed deer (OV1321) to 66 other EHDV strains. The tree was generated from the nucleotide sequence alignment of the VP3 gene. The included EHDV and BTV strains are indicated by serotype, country, state (for U.S. strains), strain or isolate name, and GenBank accession no. Nodes with black circles are supported by bootstrap values ≥ 80%. The tree was rooted with the BTV-28 strain. Additional metadata for each virus in the tree are provided in Supplemental Table S1; Figure S2: Maximum Likelihood cladogram depicting the relationships of the U.S. novel reassortant EHDV-6 strain isolated from a white-tailed deer (OV1321) to 66 other EHDV strains. The tree was generated from the nucleotide sequence alignment of the VP6 gene. The included EHDV and BTV strains are indicated by serotype, country, state (for U.S. strains), strain or isolate name, and GenBank accession no. Nodes with black circles are supported by bootstrap values ≥ 80%. The tree was rooted with the BTV-28 strain. Additional metadata for each virus in the tree are provided in Supplemental Table S1; Figure S3: Maximum Likelihood cladogram depicting the relationships of the U.S. novel reassortant EHDV-6 strain isolated from a white-tailed deer (OV1321) to 66 other EHDV strains. The tree was generated from the nucleotide sequence alignment of the NS1 gene. The included EHDV and BTV strains are indicated by serotype, country, state (for U.S. strains), strain or isolate name, and GenBank accession no. Nodes with black circles are supported by bootstrap values ≥ 80%. The tree was rooted with the BTV-28 strain. Additional metadata for each virus in the tree are provided in Supplemental Table S1; Figure S4: Maximum Likelihood cladogram depicting the relationships of the U.S. novel reassortant EHDV-6 strain isolated from a white-tailed deer (OV1321) to 66 other EHDV strains. The tree was generated from the nucleotide sequence alignment of the NS3 gene. The included EHDV and BTV strains are indicated by serotype, country, state (for U.S. strains), strain or isolate name, and GenBank accession no. Nodes with black circles are supported by bootstrap values ≥ 80%. The tree was rooted with the BTV-28 strain. Additional metadata for each virus in the tree are provided in Supplemental Table S1; Figure S5: Maximum Likelihood cladogram depicting the relationships of the U.S. novel reassortant EHDV-6 strain isolated from a white-tailed deer (OV1321) to 66 other EHDV strains. The tree was generated from the nucleotide sequence alignment of the VP5 gene. The included EHDV and BTV strains are indicated by serotype, country, state (for U.S. strains), strain or isolate name, and GenBank accession no. Nodes with black circles are supported by bootstrap values ≥ 80%. The tree was rooted with the BTV-28 strain. Additional metadata for each virus in the tree are provided in Supplemental Table S1; Figure S6: Maximum Likelihood cladogram depicting the relationships of the U.S. novel reassortant EHDV-6 strain isolated from a white-tailed deer (OV1321) to 66 other EHDV strains. The tree was generated from the nucleotide sequence alignment of the VP7 gene. The included EHDV and BTV strains are indicated by serotype, country, state (for U.S. strains), strain or isolate name, and GenBank accession no. Nodes with black circles are supported by bootstrap values ≥ 80%. The tree was rooted with the BTV-28 strain. Additional metadata for each virus in the tree are provided in Supplemental Table S1; Table S1: Serotype, strain/isolate name, country, U.S. state, year of detection, host, and GenBank accession numbers for all 10 segments of selected EHDV and BTV strains used in the phylogenetic analysis.
