# Supplementary material for: Characterization of a Novel Reassortant Epizootic Hemorrhagic Disease Virus Serotype 6 Strain Isolated from Diseased White-Tailed Deer (Odocoileus virginianus) on a Florida Farm
Source: Viruses. 2022 May 10;14(5):1012. doi: 10.3390/v14051012 (PMC9146129; doi:10.3390/v14051012)
Supplement: Supplementary file 1 [file viruses-14-01012-s001.zip › Supplemental Table S1.pdf]

**Supplemental Table S1.** Serotype, strain/isolate name, country, U.S. state, year of detection, host, and GenBank accession numbers for all 10 segments of selected EHDV and BTV strains used in the phylogenetic analysis.

| Serotype | Name | Country | U.S. State | Year | Host | Seg1-VP1 | Seg2-VP2 | Seg3-VP3 | Seg4-VP4 | Seg5-NS1 | Seg6-VP5 | Seg7-VP7 | Seg8-NS2 | Seg9-VP6 | Seg10-NS3 |
|---|---|---|---|---|---|---|---|---|---|---|---|---|---|---|---|
| EHDV6 | OV1321 | USA | FL | 2019 | WTD | OK106265 | OK106266 | OK106267 | OK106268 | OK106269 | OK106270 | OK106271 | OK106272 | OK106273 | OK106274 |
| EHDV1 | 10-0235-1 | USA | TX | 2010 | *Cervidae* sp. | KF570113 | KF570114 | KF570115 | KF570116 | KF570120 | KF570117 | KF570119 | KF570121 | KF570118 | KF570122 |
| EHDV1 | 14-04039-2 | USA | TX | 2014 | unknown | MG737824 | MG737825 | MG737826 | MG737827 | MG737831 | MG737828 | MG737830 | MG737832 | MG737829 | MG737833 |
| EHDV1 | 362B1 | USA | unknown | unknown | unknown | KU140717 | KU140741 | KU140765 | KU140789 | KU140813 | KU140837 | KU140885 | KU140861 | KU140899 | KU140933 |
| EHDV1 | CC332-06 | USA | LA | 2006 | WTD | KU140723 | KU140747 | KU140771 | KU140795 | KU140819 | KU140843 | KU140891 | KU140867 | KU140918 | KU140939 |
| EHDV1 | CC211-06 | USA | MO | 2006 | WTD | KU140722 | KU140746 | KU140770 | KU140794 | KU140818 | KU140842 | KU140890 | KU140866 | KU140917 | KU140938 |
| EHDV1 | Parker-A | USA | TX | 2008 | WTD | KU140721 | KU140745 | KU140769 | KU140793 | KU140817 | KU140841 | KU140889 | KU140865 | KU140916 | KU140937 |
| EHDV1 | E-10-4296 | USA | AL | 2010 | WTD | KU140720 | KU140744 | KU140768 | KU140792 | KU140816 | KU140840 | KU140888 | KU140864 | KU140915 | KU140936 |
| EHDV1 | E-10-4497 | USA | AL | 2010 | WTD | KU140719 | KU140743 | KU140767 | KU140791 | KU140815 | KU140839 | KU140887 | KU140863 | KU140914 | KU140935 |
| EHDV1 | IbAr22619 | Nigeria |  | 1967 | *Culicoides* spp. | AM745007 | AM745008 | AM745009 | AM745010 | AM745011 | AM745012 | AM745013 | AM745014 | AM745015 | AM745016 |
| EHDV1 | New Jersey | USA | NJ | unknown | unknown | KU140704 | KU140740 | KU140752 | KU140776 | KU140800 | KU140836 | KU140872 | KU140848 | KU140896 | KU140920 |
| EHDV1 | New Jersey | USA | NJ | 1955 | WTD | AM744977 | AM744978 | AM744979 | AM744980 | AM744981 | AM744982 | AM744983 | AM744984 | AM744985 | AM744986 |
| EHDV1 | OV202 | USA | FL | 2015 | WTD | MF688826 | MF688827 | MF688828 | MF688829 | MF688833 | MF688830 | MF688832 | MF688834 | MF688831 | MF688835 |
| EHDV1 | SV-123 | USA | NJ | unknown | unknown | KU140703 | KU140739 | KU140751 | KU140775 | KU140799 | KU140835 | KU140871 | KU140847 | KU140895 | KU140919 |
| EHDV2 | CSIRO 439 | Australia |  | 1979 | cattle | AM744987 | AM744988 | AM744989 | AM744990 | AM744991 | AM744992 | AM744993 | AM744994 | AM744995 | AM744996 |
| EHDV2 | Alberta | Canada |  | 1962 | WTD | AM744997 | AM744998 | AM744999 | AM745000 | AM745001 | AM745002 | AM745003 | AM745004 | AM745005 | AM745006 |
| EHDV2 | 13-04197 | USA | IN | 2013 | cattle | KJ125190 | KJ125191 | KJ125192 | KJ125193 | KJ125197 | KJ125194 | KJ125196 | KJ125198 | KJ125195 | KJ125199 |
| EHDV2 | 11-3174-1 | USA | PA | 2012 | *Cervidae* sp. | KF570123 | KF570124 | KF570125 | KF570126 | KF570130 | KF570127 | KF570129 | KF570131 | KF570128 | KF570132 |
| EHDV2 | CC 126-00 | USA | NC | 2000 | WTD | HM636897 | HM636898 | HM636899 | HM636900 | HM636901 | HM636902 | HM636903 | HM636904 | HM636905 | HM636906 |
| EHDV2 | 76-5460-M-32313-75 | USA | ID | 1975 | WTD | KU140715 | KU140737 | KU140763 | KU140787 | KU140811 | KU140833 | KU140883 | KU140859 | KU140897 | KU140931 |
| EHDV2 | CC211-91 | USA | WY | 1991 | unknown | KU140713 | KU140735 | KU140761 | KU140785 | KU140809 | KU140831 | KU140881 | KU140857 | KU140911 | KU140929 |
| EHDV2 | CC110-93 | USA | AL | 1993 | unknown | KU140712 | KU140734 | KU140760 | KU140784 | KU140808 | KU140830 | KU140880 | KU140856 | KU140910 | KU140928 |
| EHDV2 | CC140-93 | USA | SC | 1993 | unknown | KU140711 | KU140733 | KU140759 | KU140783 | KU140807 | KU140829 | KU140879 | KU140855 | KU140909 | KU140927 |
| EHDV2 | CC11-295 | USA | KS | 2011 | WTD | KU140706 | KU140728 | KU140754 | KU140778 | KU140802 | KU140824 | KU140874 | KU140850 | KU140904 | KU140922 |
| EHDV2 | CC12-351 | USA | MO | 2012 | WTD | KU140705 | KU140727 | KU140753 | KU140777 | KU140801 | KU140823 | KU140873 | KU140849 | KU140903 | KU140921 |
| EHDV2 | Ibaraki BK13 | Japan |  | 1997 | cattle | KM509050 | KM509051 | KM509052 | KM509053 | KM509054 | KM509055 | KM509056 | KM509057 | KM509058 | KM509059 |

| | | | | | | | | | | | | | | | |
|---|---|---|---|---|---|---|---|---|---|---|---|---|---|---|---|
| EHDV2 | No name | Italy | | 2011 | cattle | KU173874 | KU173875 | KU173876 | KU173877 | KU173878 | KU173879 | KU173880 | KU173881 | KU173882 | KU173883 |
| EHDV2 | KS-8 E 13 | Japan | | 2013 | cattle | LC599911 | LC202952 | LC202974 | LC599912 | LC599913 | LC202963 | LC599914 | LC599915 | LC599916 | LC599917 |
| EHDV2 | OV215 | USA | FL | 2015 | WTD | MF688816 | MF688817 | MF688818 | MF688819 | MF688823 | MF688820 | MF688822 | MF688824 | MF688821 | MF688825 |
| EHDV2 | OV610 | USA | FL | 2017 | WTD | MK958987 | MK958988 | MK958989 | MK958990 | MK958994 | MK958991 | MK958993 | MK958995 | MK958992 | MK958996 |
| EHDV2 | OV617 | USA | FL | 2017 | WTD | MK958997 | MK958998 | MK958999 | MK959000 | MK959004 | MK959001 | MK959003 | MK959005 | MK959002 | MK959006 |
| EHDV2 | OV862 | USA | FL | 2018 | WTD | MK959007 | MK959008 | MK959009 | MK959010 | MK959014 | MK959011 | MK959013 | MK959015 | MK959012 | MK959016 |
| EHDV2 | Cow17 | USA | MO | 1998 | cattle | MH845239 | MH845240 | MH845241 | MH845242 | MH845243 | MH845244 | MH845245 | MH845246 | MH845247 | MH845248 |
| EHDV2 | CC372-07b | USA | IN | 2012 | cattle | MH845369 | MH845370 | MH845371 | MH845372 | MH845373 | MH845374 | MH845375 | MH845376 | MH845377 | MH845378 |
| EHDV2 | 12-36567 | USA | IA | 2012 | cattle | MH845249 | MH845250 | MH845251 | MH845252 | MH845253 | MH845254 | MH845255 | MH845256 | MH845257 | MH845258 |
| EHDV2 | 12-39007 | USA | IA | 2012 | cattle | MH845299 | MH845300 | MH845301 | MH845302 | MH845303 | MH845304 | MH845305 | MH845306 | MH845307 | MH845308 |
| EHDV2 | 12-41125 | USA | MN | 2012 | cattle | MH845289 | MH845290 | MH845291 | MH845292 | MH845293 | MH845294 | MH845295 | MH845296 | MH845297 | MH845298 |
| EHDV2 | 12-35934 | USA | NE | 2012 | cattle | MH845269 | MH845270 | MH845271 | MH845272 | MH845273 | MH845274 | MH845275 | MH845276 | MH845277 | MH845278 |
| EHDV2 | 12-36542-7 | USA | NE | 2012 | cattle | MH845319 | MH845320 | MH845321 | MH845322 | MH845323 | MH845324 | MH845325 | MH845326 | MH845327 | MH845328 |
| EHDV2 | 12-36542-8 | USA | NE | 2012 | cattle | MH845329 | MH845330 | MH845331 | MH845332 | MH845333 | MH845334 | MH845335 | MH845336 | MH845337 | MH845338 |
| EHDV2 | 12NE-0024A | USA | NE | 2012 | cattle | MH845279 | MH845280 | MH845281 | MH845282 | MH845283 | MH845284 | MH845285 | MH845286 | MH845287 | MH845288 |
| EHDV2 | 12-43618 | USA | OH | 2012 | cattle | MH845379 | MH845380 | MH845381 | MH845382 | MH845383 | MH845384 | MH845385 | MH845386 | MH845387 | MH845388 |
| EHDV2 | 12-035472-4 | USA | SD | 2012 | cattle | MH845259 | MH845260 | MH845261 | MH845262 | MH845263 | MH845264 | MH845265 | MH845266 | MH845267 | MH845268 |
| EHDV2 | 12-15085 | USA | SD | 2012 | cattle | MH845309 | MH845310 | MH845311 | MH845312 | MH845313 | MH845314 | MH845315 | MH845316 | MH845317 | MH845318 |
| EHDV2 | 12-150871 | USA | SD | 2012 | cattle | MH845339 | MH845340 | MH845341 | MH845342 | MH845343 | MH845344 | MH845345 | MH845346 | MH845347 | MH845348 |
| EHDV2 | 12-39713 | USA | SD | 2012 | bison | MH845349 | MH845350 | MH845351 | MH845352 | MH845353 | MH845354 | MH845355 | MH845356 | MH845357 | MH845358 |
| EHDV2 | GA | USA | GA | 2016 | WTD | MN824457 | MN824458 | MN824459 | MN824460 | MN824461 | MN824462 | MN824463 | MN824464 | MN824465 | MN824466 |
| EHDV4 | IbAr 33853 | Nigeria | | 1968 | *Culicoides* spp. | AM745017 | AM745018 | AM745019 | AM745020 | AM745021 | AM745022 | AM745023 | AM745024 | AM745025 | AM745026 |
| EHDV5 | CSIRO 157 | Australia | | 1977 | cattle | AM745027 | AM745028 | AM745029 | AM745030 | AM745031 | AM745032 | AM745033 | AM745034 | AM745035 | AM745036 |
| EHDV6 | TAT2013/02 | Trinidad | | 2013 | cattle | MK919254 | MK919255 | MK919256 | MK919257 | MK919258 | MK919259 | MK919260 | MK919261 | MK919262 | MK919263 |
| EHDV6 | 318 | Bahrain | | 1983 | cattle | AM745067 | AM745068 | AM745069 | AM745070 | AM745071 | AM745072 | AM745073 | AM745074 | AM745075 | AM745076 |
| EHDV6 | 12-3437-8 | USA | OH | 2012 | *Cervidae* sp. | KF570133 | KF570134 | KF570135 | KF570136 | KF570140 | KF570137 | KF570139 | KF570141 | KF570138 | KF570142 |
| EHDV6 | CC304-06 | USA | IN | 2006 | WTD | HM641772 | HM641773 | HM641774 | HM641775 | HM641776 | HM641777 | HM641778 | HM641779 | HM641780 | HM641781 |
| EHDV6 | CSIRO 753 | Australia | | 1981 | cattle | AM745037 | AM745038 | AM745039 | AM745040 | AM745041 | AM745042 | AM745043 | AM745044 | AM745045 | AM745046 |
| EHDV6 | M44 96 | South Africa | | 1996 | cattle | HM636907 | HM636908 | HM636909 | HM636910 | HM636911 | HM636912 | HM636913 | HM636914 | HM636915 | HM636916 |
| EHDV6 | OV208 | USA | FL | 2016 | WTD | MG886400 | MG886401 | MG886402 | MG886403 | MG886407 | MG886404 | MG886406 | MG886408 | MG886405 | MG886409 |

| | | | | | | | | | | | | | | | |
|---|---|---|---|---|---|---|---|---|---|---|---|---|---|---|---|
| EHDV6 | 12-38993-2 | USA | IL | 2012 | cattle | MH845389 | MH845390 | MH845391 | MH845392 | MH845393 | MH845394 | MH845395 | MH845396 | MH845397 | MH845398 |
| EHDV7 | CSIRO 775 | Australia | | 1981 | cattle | AM745047 | AM745048 | AM745049 | AM745050 | AM745051 | AM745052 | AM745053 | AM745054 | AM745055 | AM745056 |
| EHDV7 | FO-1 E 16 | Japan | | 2016 | cattle | LC599904 | LC552731 | LC552732 | LC599905 | LC599906 | LC552733 | LC599907 | LC599908 | LC599909 | LC599910 |
| EHDV7 | ISR2006 02 | Israel | | 2006 | cattle | KM391743 | KM391727 | KM391737 | KM391751 | KM391725 | KM391753 | KM391739 | KM391745 | KM391736 | KM391732 |
| EHDV7 | ISR2006 04 | Israel | | 2006 | cattle | KM391733 | KM391729 | KM391749 | KM391728 | KM391752 | KM391726 | KM391750 | KM391735 | KM391738 | KM391731 |
| EHDV7 | ISR2006 06 | Israel | | 2006 | cattle | KM391741 | KM391746 | KM391740 | KM391742 | KM391744 | KM391734 | KM391748 | KM391730 | KM391747 | KM391724 |
| EHDV7 | ISR2006 | Israel | | 2006 | cattle | JQ070177 | JQ070178 | JQ070179 | JQ070180 | JQ070181 | JQ070182 | JQ070183 | JQ070184 | JQ070185 | JQ070186 |
| EHDV7 | YN09-04 | China | | 2013 | cattle | MK656453 | MK656454 | MK656455 | MK656456 | MK656457 | MK656458 | MK656459 | MK656460 | MK656461 | MK656462 |
| EHDV8 | CPR 3961A | Australia | | 1982 | cattle | AM745057 | AM745058 | AM745059 | AM745060 | AM745061 | AM745062 | AM745063 | AM745064 | AM745065 | AM745066 |
| EHDV | JC13C644 | China | | 2013 | *Culicoides* spp. | MT013324 | MT013325 | MT013326 | MT013327 | MT013328 | MT013329 | MT013330 | MT013331 | MT013332 | MT013333 |
| EHDV | JC13C673 | China | | 2013 | *Culicoides* spp. | MT013314 | MT013315 | MT013316 | MT013317 | MT013318 | MT013319 | MT013320 | MT013321 | MT013322 | MT013323 |
| BTV28 | 1537 | Israel | | 2014 | sheep | MH559813 | MH559807 | MH559808 | MH559814 | MH559812 | MH559815 | MH559811 | MH559810 | MH559816 | MH559809 |

WTD: white-tailed deer.
